# Blogging as a Tool for Real-Time Learning in Medical Microbiology

**DOI:** 10.3389/fmicb.2021.576145

**Published:** 2021-03-04

**Authors:** Charmaine Lloyd

**Affiliations:** ^1^School of Life Sciences & Chemical Technology, Ngee Ann Polytechnic, Singapore, Singapore; ^2^School of Health Sciences, Swinburne University of Technology, Melbourne, VIC, Australia

**Keywords:** blog, microbiology, e-learning, engaged learning, disease tracking, social media, edublog, infectious disease

## Abstract

Blogging is a widely used social medium for storing and sharing information online. Being an attractive online interface, some studies show that education blogging or edublogging might promote more engaged learning. An apathy to contemporary issues related to one’s area of study can result in a less knowledgeable student who is less ready for the job industry. To bridge the gap between classroom learning and awareness of emerging issues pertaining to the field of study and potential employment – blogging of ongoing events in a select microbiological field was proposed as a graded semester-long activity called “Disease Tracking.” The exercise involved instructing students to choose one infectious disease topic, for which traditional and non-traditional scientific information could be sourced with high frequency over the preceding months. Students were to document new information on the topic as it became available over the term, from reliable information resources. At the end of the term, students presented their work in a “Blog show-off” presentation session. Blog-based learning was found to be an engaging tool that satisfied all criteria under Bloom’s taxonomy. Students developed a continued intrigue for the chosen topic and appreciated the diverse fields in which fundamentals of infectious diseases learned in class, could be applied within and outside academia. Students also valued this experience and feedback showed that the freedom to choose their own topic (77%), opportunity to learn more from other students’ blogs (77%), less stress as they were not competing on identical topics (73%), a “fun way” to learn (68%), and an opportunity to understand the importance of staying abreast with scientific news (64%) stood out as the chief positive points of the exercise to the students. In view of these benefits, blogs can be used for an immersive, broad learning experience in Microbiology and other fields in which there is likely to be a frequency of new information online.

## Introduction

Undergraduate education in long-evolved sciences such as Microbiology (which is the focus of this education research issue) is challenging even for a passionate student, due to the vast content and unfamiliar terminologies (Struwig et al., 2016). Traditional methods of teaching microbiology such as theory and practicals may result solely in the recollection of facts and skills for exams. Activities that could stimulate reasoning (Patil and Karadesai, 2016) or learning beyond the classroom often take a back-seat due to time constraints. Student-led scientific presentations, journal club discussions, classes, and seminars (Spruijt et al., 2013; Dinkel, 2020) are useful, yet sometimes perceived as boring and heavy tasks (Stuart, 2013). Board games, gaming, and project/case-based learning (Beyfield and Struwig, 2007; Struwig et al., 2014; Mateo and Sevillano, 2018) have been attempted by educators with varying success to reinforce fundamentals. Some educators have even sought the use of social networking such as Whatsapp and Facebook to keep students engaged on case studies, whereas others have reported students to perceive this as sometimes intrusive (Hershkovitz et al., 2019; Van Den Beemt et al., 2020). It is interesting to note that while students are heavily engrossed in social platforms (Abbas et al., 2019), not many have an appetite for trending world events or scientific news (Crane and Cox, 2013; Medrano, 2014). While learning of traditional knowledge and skills are important, students also need to stay abreast with world events pertaining to their chosen discipline of study. Discussion boards on learning management systems (LMS) might be used to promote this. Often, a lack of appeal of the interface, absence of immediate feedback, and poor use of interactive tools by the educator (Ramayah and Lee, 2012) result in lowered students’ interest.

Blogging is not new to our tech-savvy undergraduates. They use blogs to record their experiences in life and learning (Zhang and Olfman, 2010); for personal/study reflections, as a repository for learning materials (Yang, 2009; Ginani et al., 2012) and a ready source of information for online communities (Jolly and Matthews, 2017). Why blogs over other social media? Undergraduates perceive blogs as an exciting way to share their “newly learned scholarly knowledge” from a single portal with their networks (Lowe et al., 2016), while also reaping social benefits of networking, such as the gratification in social enhancement, recognition, and appreciation (Srivastava et al., 2019). Educators from a variety of fields have used blogs for collaboration and enhancing learning – for example, marketing blogs, ICT, and industry project development (Luca and McLoughlin, 2005a; Duarte, 2015).

Polytechnic undergraduates in Biomedical Science are required to be job-ready with laboratory skills and strong subject knowledge. If efforts are taken to encourage students into being conversant with scientific affairs beyond the classroom, it may help them make informed choices about their career. In this article, I share my perspective with some examples, as to why blogging on microbiology in the news over a term as a graded exercise, might be an engaging and stimulating learning tool for both the students and the educator.

## Disease Tracking for Edublogging in Microbiology

The blogging activity titled “Disease tracking” (DT) was conducted under the medical microbiology module for second year Biomedical science diploma students at Ngee Ann Polytechnic between 2015 and 2018. The module ran for a semester (approximately 4 months). The pre-requisite for this module was a pass in year 1 General Microbiology.

### Implementing Blogging and Learning Objectives

The major learning objectives of this exercise was (a) to give students an opportunity to connect foundational knowledge and laboratory techniques learned in the class with current relevant topics published in the field and (b) to ignite an interest in students to look beyond social media and engage in learning from scientific resources.

In week 1, the class collaborated at a tutorial to perform an online search for infectious diseases that appeared frequently in national or international news sites. Examples of these diseases could be frequent articles on an outbreak, clinical trial, antibiotic resistance issues, etc. The topics once finalized, were listed on a shared online document that students used to choose their disease of interest. If this is to be adapted to a larger cohort or undertaken subsequently, then depending on the class size and the number of diseases trending for that span of time, an individual or a team of student blogger(s) could take on a single topic. Students created the blogging site using any of the freely available online blogging tools and the web address was shared to the class using the virtual learning environment. In our case, this was Blackboard. For future cohorts or in different institutions, other Learning Environments would also work.

### Expectations

The “Disease Tracking” blog was to be a chronological documentation of a chosen trending infectious disease during the term of study. Articles if sparse could be sourced from 6 months to a year in advance and followed up thereafter. While an introduction was permissible, the blog was not to be a lesson/seminar about the microorganism. It was intended to document every source of information about that infectious disease. The resources could range from but were not limited to – news and scientific news articles; information from Centre for Disease Control (CDC), United States, WHO, Ministry of Health Singapore; and articles from PubMed, research centers, and clinical trial sites. Students were to blog about the articles in their own words, appropriately referencing the sources and avoiding plagiarism. An end-of-term “Blog Show Off” was conducted to promote sharing of the content collected over the term. The whole DT exercise accounted for 15% of the overall grade. Marks were awarded by the lecturer/instructor/teacher, under the following categories: contributions per member, a good distribution of article resources, a proper simplified interpretation, ability to explain the article verbally at the end of presentation (to ensure it was not transcribed without understanding), good presentation skills, and being able to connect back to the fundamentals learned from lectures. A short feedback survey was conducted to understand students’ opinions of the blogging exercise. This is discussed in the next section.

## Discussion

Some of the examples of the DT blogs prepared by the polytechnic students are tabulated in Table 1 (provided with permission from students). Within the 12 weeks of a semester, students had to contribute to a minimum of eight posts. The blogs should be seen as a testament to students’ abilities to explore subject information and creatively document relevant contemporary events in a study discipline beyond the limits of the curriculum. While this is also possible with journal and seminar preparation and presentation, blogging has the added potential to encourage interaction while it is being developed between blogging groups (Ferdig and Trammel, 2004; Duarte, 2015).

**Table 1:** Examples of students’ blogs on disease tracking.

Name of student’s blog	Link to blog
Rabies (Low, 2015)	http://rabiesdiseasetracking2015.blogspot.com/
Mersecode (Tan, 2016)	https://mersecode.wordpress.com/
Dengue (Teo, 2017)	https://onceyougodengueyouwontbehealthy.wordpress.com/about/
Human immunodeficiency virus (Song et al., 2018)	https://humanimmunodeficiencyvirusnpbms.wordpress.com/page/2/
Cholera (Ang and Beh, 2018)	https://cholerabms.wordpress.com/

Students’ impressions of the positive points in the blogging exercise have been tabulated (Table 2). Out of 28 students in the cohort, 22 responders shared their feedback. More than half the respondents agreed that the top reasons why they appreciated the “Disease tracking” blogging exercise was because they got to choose their own topic (77%), they were learning more from other students’ blogs (77%), they were not in competition with each other on identical topics (73%), found it a fun way to learn (68%), and understood the importance of staying abreast with scientific news (64%). Less than half the respondents thought that blogging promoted community awareness (46%), or that it was slow paced (36%), or that they learned epidemiology from the exercise (36%). Being acknowledged by fellow readers (27%), being peer reviewed by classmates after presentation or having a blog that was uniquely theirs (27%) mattered less as a positive point. Understandably, only 23% felt more confident through this exercise to research and understand complex papers on the microorganism being blogged (out of increased interest).

**Table 2:** Student’s impressions on edublogging for learning in the “disease tracking” exercise.

Characteristics of the “disease tracking” blog exercise	Students in agreement with the characteristic *n* = 22 (%)
Freedom to choose student’s/team’s own topic	17 (77%)
Opportunity to learn more from other students’/teams’ blogs	17 (77%)
Less stressful as students were not competing on identical topics	16 (73%)
A “fun way” to learn	15 (68%)
Opportunity to understand the importance of staying abreast with scientific news	14 (64%)
Occasion to learn the importance of blogging in community awareness	10 (46%)
Ownership of a piece of online compilation	9 (41%)
Slow paced nature of the exercise	8 (36%)
Opportunity to learn the epidemiological distributions of disease	8 (36%)
Being acknowledged by fellow readers	6 (27%)
Being peer reviewed by class mates after their presentation	6 (27%)
Presenting a blog that was uniquely their’s	6 (27%)
Built confidence to understand complex papers on the microorganism and infectious disease being blogged.	5 (23%)

Understanding the views of students toward this blogging exercise was important and feedback was sought. Written feedback provided by the students also showed that they found this a worthwhile and interesting approach to learning about infectious diseases. Comments included: “very unique educational approach,” “less of an obligation, but an interest,” “more useful and interesting than doing presentations on PubMed articles,” “unique way of presenting information to the class, instead of using PowerPoint,” “not done solely just for grades but for learning itself – knowledge I have gained from blogging will be remembered even after this module has ended!,” “Having sat through the sharing seasons by my classmates, I feel like I’ve really taken a lot home, and it felt really rewarding.” One respondent felt that “it was quite tough” to incorporate in their busy schedule. The majority (20/22) respondents agreed that they appreciated the importance of the subject better. All respondents recommended this learning method for future cohorts.

As described by other educators (Ferdig and Trammel, 2004; Schroeder et al., 2010), blogging provided a flexibility in teaching and learning and promoted a collaborative knowledge construction. The experience of having a class of engaged listeners through a blog presentation exercise was rewarding. Students were curious about the various snippets of information. The mix of news articles, scientific articles, government policies, international body, and public opinions was able to capture the attention of all listeners. The dynamic variety of colorful blog interfaces when swiped through contributed to visual appeal. It was an eye-opening experience for young polytechnic students to envisage how a fundamental class chapter, for example –antibiotic resistance, had far reaching connections.

As the blog contributed to the students’ final grade, the blogging exercise was always completed. Reluctance to blog (Duarte, 2015) by some, led to shabby blogs that were populated close to the deadline. The challenge of discerning fair and equitable work in groupwork (Luca and McLoughlin, 2005b) was not difficult with blogs, as it usually is with other group assignments, as every blog post is accompanied by a time-stamp against the contributor’s name. From an education point of view, the exercise of blogging to learn about a trending disease, ticked all the boxes in the Bloom’s taxonomy (Bloom, 1956) checklist. The “creativity” and quality of presentation in the blogs were appealing. Skills in interpreting scientific articles evolved, sometimes with a journalistic flair! Students were able to “evaluate” and “analyze” the extent, burden, and management of disease spread over time, from a wide variety of perspectives (medical, scientific, community, and social). They began to formulate their own views on how the health system was run and began to question policies. It was observed that students were also able to “apply” their understanding garnered from lectures and practicals directly into the “whats and whys” of diagnostic tests, outbreaks, experiments, vaccines, and clinical trials. They were able to “understand” the purpose of learning historical snippets in Microbiology. Students appreciated how knowledge is built up over time/generations and how this knowledge goes into the identification of etiological agents in outbreaks, disease eradication, immunization, the role of community, and the value of strong leadership in such instances. The blog approach caused students to revisit their fundamentals several times, thus helping them to “remember” their basics better.

The role of the lecturer was to commend contributors, stimulate scholarly discussions, and thus gently stimulate more contributions. Students met with additional concepts in immunology, biosecurity, and epidemiology along their learning journey. They were able to appreciate the interdisciplinary approaches taken to study and control infectious diseases.

The advantage of using a blog in microbiology is that there is no dearth of reports of infectious diseases to source from. Blogging about various diseases post-Covid-19 will be important to refocus on the many forgotten but prevalent diseases. Students may also share their blog link in their resumes. Some of the disadvantages in the current experience is that (a) if students did not use their actual names in the blogs, they may be acknowledged only by their user name in citations, (b) blogs cannot be submitted through plagiarism software for checking, (c) in the rare case, a student may decide to take down their blog and it cannot be referenced anymore, and (d) students may or may not decide to continue populating the blog after the course.

A similar blogging exercise can be recommended for any subject, where there is likely to be frequent resources of new information.

## Conclusion

Blogging about even one disease over one academic term broadened a student’s outlook to the myriad scenarios in which microbiology is applied, within and outside academia. This is a good approach to extend the curriculum beyond the classroom. Students become active and independent learners. They see for themselves the various infectious disease problems and gaps in medical management. This spurs them to delve deeper into the subject, ask questions, and become potential problem solvers (Lujan and DiCarlo, 2006) from an early stage in their career.

## Data Availability Statement

The raw data supporting the conclusions of this article will be made available by the authors, without undue reservation.

## Ethics Statement

Ethical review and approval was not required for the study on human participants in accordance with the local legislation and institutional requirements. Written informed consent from the participants’ legal guardian/next of kin was not required to participate in this study in accordance with the national legislation and the institutional requirements.

## Author Contributions

The entire work published here-in is the teaching pedagogy designed, executed, and reviewed by the sole author.

### Conflict of Interest

The author declares that the research was conducted in the absence of any commercial or financial relationships that could be construed as a potential conflict of interest.
